# Fractures of the Nose1Read before the Section on Ophthalmology, Otology, Rhinology and Laryngology, Buffalo Academy of Medicine, March 18, 1901.

**Published:** 1901-06

**Authors:** Marshall Clinton

**Affiliations:** Buffalo, N. Y.; Attending Surgeon Erie County Hospital, Assistant Attending Surgeon Sisters’ and Buffalo Children’s Hospitals, Etc.


### FRACTURES OF THE NOSE.[1]

By MARSHALL CLINTON, M. D., Buffalo, N. Y.

Attending Surgeon Erie County Hospital, Assistant Attending Surgeon Sisters' and Buffalo Children's Hospitals, Etc.

THE nose, besides being the special organ of the sense of smell, is also one of the organs of facial beauty; the one which of all others detracts from an ideal of facial symmetry when it has suffered injury and deformity.

In the consideration of injuries and deformities a study of the anatomy of the nose is necessary, and we are struck by certain well-known, but none the less, interesting facts. Two-thirds of the facial portion, and one-third of the septum, are made up of cartilage, while the rest is made up of the bony structures forming the nasal, vomer and ethmoid.

The purely disfiguring deformities are found in the cartilaginous framework, which makes up most of the facial portion, and this structure is worthy of some remark. Two lateral, triangular cartilages are found on either side attached to the nasal bones above—the superior maxillæ behind, and to the septum and one from the opposite side in front. The cartilages are rather brittle, thinner in the middle than at their attachments, and united to the bones by connective tissue. This gives us a delicate, yielding framework, solidly attached above and open below. Motion is free in lateral directions, slight in an upward, and less downward, when applied to the tip. When, however, any force is applied to the root very slight motion is permitted in any direction. The delicate way in which the upper lateral and septal cartilages are attached to the contiguous bony parts is of note, for in no other part of the body is found an osteochondral articulation with the edge of the cartilage attached to the bone, with simply the periosteum of the bone serving as the attaching ligaments.

From a cosmetic point of view we can disregard injuries to the bony portion of the nose. There is one anatomical point in connection with the fossæ which is well to remember—namely, the anastomosis of the nasal veins with the veins within the cranium through the cribiform plate of the ethmoid. The lymphatics communicate with the beginning posterior chain of the cervical and retropharyngeal. Any severe force applied to the nose will be transmitted in two directions—down, in which case the force acts on the superior maxilla and vomer; upward, and the force acts on the nasal bones and the ethmoid. The nasal bones being the most prominent, get, therefore, of

1. Read before the Section on Ophthalmology, Otology, Rhinology and Laryngology, Buffalo Academy of Medicine, March 18, 1901.

all the bony structures, the greatest amount of damage to sustain. The ethmoid, while it articulates with thirteen bones, derives its chief support from the sphenoidal and frontal bones. To these, then, are transmitted the force of impact affecting the ethmoid.

The cause of all fractures of the nose is direct violence. In the analysis of about fifty cases we find that a fracture-dislocation of one of the three main cartilages is the commonest injury. By the main cartilages I mean the septal and two upper lateral. Any force severe enough to flatten the nose will cause the fibrous portions of the cartilages to rupture and a dislocation or dislocation-fracture results. In true fractures of a cartilage, we find an angular deformity, as in green stick fracture of bone and not an over-riding of fragments from muscular pull, as in a complete fracture in bone. The fragments in injury to the lateral cartilages are found to rotate, so that the upper border will be found below the nasal bones, and the lower portion projecting either outward or inward; depending on the direction of the line of fracture. This same deformity occurs in dislocation of the lateral cartilages, and for all practical purposes we can regard them as the same injury. In fracture or dislocation of the septal cartilage, we have a rotation to one side of the superior border and usually a partial fracture of the middle portion, which projects into one of the nasal fossæ. This deformity causes the common twisted nose, as the change in the vertical axis of the septal cartilage changes the direction that the nose points. When the line of fracture in the septal cartilage is through its entire length and the angle is great enough to allow a depression of the upper border, we find a falling in of the two lateral cartilages and a flat nose as a result. This condition is dependent on two conditions, and the proper recognition of the prevailing one is necessary for correct treatment. It may be due to the fracture of the septal cartilage or to a dislocation and rotation downward of both lateral cartilages. Reduction will be much easier in the first instance than in the latter.

*Diagnosis*—These conditions are usually obscured to a large extent by the large amount of swelling which occurs with slight injury to the external parts of the face, and the patients are none too willing to allow of enough manipulation to discover the exact condition. Swabbing out the nose, after hemorrhage is checked, with a 2 per cent. solution of cocaine is undoubtedly the greatest help we have in arriving at a correct diagnosis. Inspection, both intranasal and extranasal, will tell us a good deal. Taking the root and body of the nose firmly between the fingers will give a sense of unusual motility when a cartilage is out of place, and when fractured the cartilaginous crepitus is quite easily detected. Intranasal inspection will show the septal cartilage bulging into one fossa quite or partly obstructing the nasal fossa. In some cases it is necessary to wait until the swelling has begun to go down before anything can be made out of a case, and here we can leave things alone for three or four days without much harm.

*Treatment*—When the condition has been recognised, correct it as soon as possible, for permanent deformity may result in dislocations by waiting until new tissue has begun to form between the dislocated borders of a cartilage. In fractures, time is not so essential, as deformity may be corrected almost any time, preferably right after an injury or within three or four days. Any small, smooth instrument in the nature of an elevator will serve to reduce dislocations or fractures of the nasal cartilages, and in their reduction always work from the inside of the nose. In correcting any deformity with an elevator it is best to over-correct, and especially when an external dressing is applied. The rather elaborate adjuvants for the treatment of these conditions, as recommended by some authors, are hardly necessary, for a careful plugging of a nostril with or without an external dressing will hold cartilages in their place. Within a few days after an injury the cartilages will be found to hold in their normal position and, except in the case of young children with downward dislocation and outward rotation of the lateral cartilages, they need only careful watching.

The sequelæ of these conditions when poor results are obtained, are merely a destruction of the symmetry of the nose, which is one of the commonest and most unsightly of cosmetic errors. The size of the nose is of slight importance so long as it is symmetrical.

Fractures of the bony portions of the nose and nasal fossæ present a more serious condition of affairs to deal with. These fractures are usually compound, and being compound deserve the consideration that compound fractures in other portions of the body are granted.

Fractures of the nasal bones are followed by a large amount of swelling and usually by emphysema of the neighboring soft tissues. The hemorrhage is profuse and troublesome at times. The resulting deformity in fractures of the nasal bones can be reduced by external manipulation.

Fractures of the vomer are rare, except in crushing injuries of the face, when they are too minor a thing to consider. Fractures of the ethmoid are more common and fractures of the perpendicular plate are seen in connection with fractures of the nasal bones. They produce no deformity and require treatment only when they give symptoms of obstruction to a fossa. The danger in these conditions is, however, from infection of a virulent type forming a thrombophlebitis which will communicate with the veins which enter the cranium through the cribriform plate. While this condition may be a rare one, yet it should always be guarded against and a case considered to be septic until convalescence is established. Caries of nasal bones, vomer or ethmoid, may result from an injury. One patient under my care last summer presented himself with an extensive periostitis of both superior maxillæ and nasal bones, following the kick of a horse some months previous. The ethmoid may be driven into the anterior fossa of the skull, and this constitutes the severest injury we have to deal with under this heading. I have seen but one case, which I report:

G. F. was struck by a railroad train running at high speed and thrown several yards. He was brought to the hospital, and it being evening at the time he was injured, the ambulance surgeon who first attended him applied bandages and dressings to a bloody face with a hazy idea of what he was dealing with. On entrance to the hospital I found the entire left side of his face turned outward and bandaged over his left ear, with the left nasal bone and left side of his nose and upper lip as the furthermost portion of the flap made by the injury. He was partially conscious and in great pain, so was anesthetised. The vomer was found to be crushed and the perpendicular plate of the ethmoid shattered. On removal of fragments, fractures were seen to extend up to the cribriform plate. Removal of spicules demonstrated the cribriform plate driven into the anterior fossa and pressing on the frontal lobes. I removed enough to admit the tip of the little finger into the anterior fossa of the skull and pulled down the parts driven upward. The whole surface was very carefully cleansed and every bit of dirt and loose particles of bone removed. The cavity, where the left side of the nose ought to have been, was filled with gauze and left in for three days. Fortunately for him, the dural surface healed without infection and after a few days he was convalescent. The cerebral disturbance was marked in the first few days, but his recovery from this rather severe injury was otherwise normal. Two years later I saw this patient, and aside from a deformity due to loss and depression of parts of the left side of his nose, he was all right.

Lacerations associated with fractured noses are at times troublesome to deal with, for no matter how carefully we approximate the wound edges, the motility of the face will allow of enough play on the edges of the wound, to make a broad scar instead of what we would like—a narrow one. Another interesting case of fractured nose was one associated with occlusion of the lachrymal duct. It required patient sounding before the obstruction was overcome, and the patient was instructed in the use of the probe and advised to use it every two weeks for the next six months.

In summing up the whole subject I would say, recognise and treat your deformities as soon after an injury as you can get hold of them, and in correcting them over-correct them for the first few days. By so doing you will avoid many cases of deformed nose, which are not a credit to any medical man called to treat these conditions.

466 FRANKLIN STREET.

